# Clinical Decision Making: Acute Ischemic Stroke with Large Vessel Occlusion

**DOI:** 10.5070/M5.60864

**Published:** 2026-07-31

**Authors:** Jennifer Yee, Christopher E San Miguel, Jennifer M Mitzman, Simiao Li-Sauerwine, Geremiha Emerson, Cynthia Leung, Krystin N Miller, Kelsey H Jordan, Sorabh Khandelwal

**Affiliations:** * The Ohio State University College of Medicine, Department of Emergency Medicine, Columbus, Ohio

## Abstract

**Audience:**

This clinical decision-making (CDM) case is intended for emergency medicine (EM) residents of all levels.

**Introduction:**

About 700,000 patients every year in the United States experience an acute ischemic stroke.^1^ Morbidity and mortality from an ischemic stroke may be limited from timely diagnosis and acute management, including specialist involvement, appropriate blood pressure control, and evaluation for possible thrombolytic therapy. EM residents should be familiar with stroke management principles whether they work in an academic, community, or rural setting.

**Educational Objectives:**

By the end of this clinical decision-making case, learners will be able to: 1) demonstrate familiarity with the CDM case format and case play, 2) describe important historical information to obtain when suspecting an acute stroke, 3) outline key diagnostic tests to rule out other causes of acute neurologic deficits, 4) outline acute management strategies for acute ischemic stroke, 5) propose an appropriate disposition plan for patients with an acute ischemic stroke.

**Educational Methods:**

This is a clinical decision-making boards case as outlined by the American Board of Emergency Medicine (ABEM). Each learner was paired with one instructor for the case, a scoring checklist by the instructor was used, structured debriefing time was incorporated into the assessment, and learners were given the opportunity to provide qualitative feedback after the case.

**Research Methods:**

Each CDM case session lasted approximately 25 minutes, with 15 minutes for the case and 10 minutes for debriefing and feedback. A 26-point critical action checklist was developed to evaluate each learner’s performance, with each point reflecting an equally weighted item. Learners then provided verbal feedback on the cases to the examiners at the conclusion of their assessments.

**Results:**

Thirty-nine categorical emergency medicine residents participated as learners for this clinical decision-making session, including 10 third-year residents, 12 second-year residents and 17 first-year residents. The average overall score was 17.3 of 26 possible points. Performance with respect to postgraduate year (PGY) is as follows: 17.7 for PGY-3s, 18.8 for PGY-2s, and 15.9 for PGY-1s. One resident had a perfect score.

**Discussion:**

Performance of our learners varied and unexpectedly, our second-year residents outperformed our third-year residents. This is likely due to our PGY-2 learners being responsible for the primary care of stroke patients in our department, which makes their identification and management of acute ischemic stroke patients likely more recently retrievable.

**Topics:**

Emergency medicine, neurology, acute ischemic stroke.

## USER GUIDE

**Table:** 

List of Resources:	
Abstract	1
User Guide	3
For Examiner Only	5
Certifying Exam Assessment	11
Stimulus	13
Debriefing and Evaluation Pearls	19

**Learner Audience:**

This CDM is intended for interns and junior and senior residents.

**Time Required for Implementation:**

Case: Clinical decision-making cases are 15 minutes by American Board of Emergency Medicine (ABEM) standard. Debriefing: 5–10 minutes per case.

**Recommended number of learners per instructor:**

It is recommended that the instructors facilitate the case one-on-one with learners. For this assessment, a mock exam day was held where multiple facilitators ran the same case repeatedly with different residents.

**Topics:**

Emergency medicine, neurology, acute ischemic stroke.

**Objectives:**

By the end of this CDM case, learners will be able to:

1. Demonstrate familiarity with the CDM case format and case play.
2. Describe important historical information to obtain when suspecting an acute stroke.
3. Outline key diagnostic tests to rule out other causes of acute neurologic deficits.
4. Outline acute management strategies for acute ischemic stroke.
5. Propose an appropriate disposition plan for patients with an acute ischemic stroke.

### Linked objectives, methods and results

By participating in this clinical decision-making case, residents have the opportunity to practice assessing a patient with an acute ischemic stroke (objectives 1–3). This scenario outlines a clinical patient case from history to exam, management, and disposition (objective 1). Historical elements such as “last known well” time, anticoagulation use, and past medical history are emphasized (objective 2), as well as completion of the National Institution of Health Stroke Scale (NIHSS) for physical exam (objective 3). Residents will be evaluated on their appropriate management, including blood pressure management and administration of thrombolytics (objective 4) with ultimate disposition to a critical care unit (objective 5) of their patient. Additionally, a built-in debriefing session at the end of the case provided real-time feedback to learners to ensure an “assessment for learning” component to the exercise.

### Recommended pre-reading for instructor

- American Board of Emergency Medicine. Sample Case: Clinical Decision Making. YouTube. Published November 27, 2024. Accessed November 23, 2025. https://www.youtube.com/watch?v=Dv_ga0Ei7oY
- American Board of Emergency Medicine Certifying Exam Case Materials: Clinical Decision Making. Published November 2024. Accessed November 23, 2025. Case-Materials_Clinical-Decision-Making.pdf

### Results and tips for successful implementation

This clinical decision-making case was created by Emergency Medicine (EM) faculty. This case was best implemented as a CDM case format (or what was formerly known as a structured interview). All 39 learners were categorical EM residents (10 third-year residents, 12 second-year residents and 17 first-year residents). Each learner was allotted 15 minutes for the case and 5–10 minutes for a debriefing afterwards. Instructors graded each learner on a 26-point grading sheet. Learners provided verbal feedback to their case facilitators during the debriefing period. Since original pilot data, the questions listed here were made more specific and updated to better reflect the new Certifying Exam based on evolving ABEM information.

The average score for all learners was 17.3 out of 26 total items. The averages for each post graduate year (PGY) class were as follows: 17.7 for PGY-3s, 18.8 for PGY-2s, and 15.9 for PGY-1s. This numerical data represents our institutional iteration of our initial understanding of the Clinical Decision-Making Cases.

The initial 26-point grading sheet used during the original assessment was updated to include an additional rationale prompt for the history section and “sign out” action for transition of care section criteria in this manuscript. Other changes made to this case for subsequent use included: expanded differential diagnoses, clarification edits to one historical question, and adding a range of acceptable blood pressure goals.

If neurology was consulted, the examiner told the resident that they are with an unstable patient upstairs and cannot come down to the emergency department at this time. This was to prevent any deferral of decisions regarding thrombolytics to the neurologic team. It is recognized that different residency programs may need to transfer for neurosurgical intervention or may not have a neuro-interventionalist in-house, so there were no mandatory items requiring discussion with this specialist.

As a result of faculty and resident feedback, historical information was described from the partner’s perspective, for if the patient themselves gave the history, residents appropriately assumed that the airway was intact and did not need to be clarified during the physical exam.

Multiple residents did not write down the patient’s vital signs, despite having sheets of paper to take notes. During debriefing, they relayed that they forgot the patient was significantly hypertensive, and therefore, did not order antihypertensive medications. Other examiners may choose to leave the vital signs available for the duration of the case or to emphasize the importance of taking notes during the examination.

At our institution, during residency assessments, understanding underlying pathophysiology principles is emphasized. This is why we specified that the rationale for thrombolytics was more detailed than “to bust up the clot” as an instructor prompt during the scenario.

Outlier items were reviewed (where residents all scored very high or if the score was lower compared to other items) to determine a) if this is an appropriately written item, and if so, b) lower-scoring items will be used as learning opportunities to emphasize within future didactics sessions. One of the lower-scoring items was a physical exam point for “assessing the airway,” which may be attributed to the chief complaint being “weakness” and not “unresponsiveness.” A second lower-scoring item was to describe the rationale for providing thrombolytics, since most residents verbalized this was to dissolve the pre-existing clot rather than to re-perfuse ischemic, at-risk tissue. The airway item has been changed here for this manuscript for this reason.

Verbal feedback from our learners primarily focused on widening our accepted differential diagnoses for future iterations. This case is appropriate for all levels of learning, particularly when a formative approach (assessment for learning) is used. Given the feedback learners and instructors provided, this case has high impact value in reviewing diagnosis and management of acute neurologic pathologies.

## Figures and Tables

**Table:** 

I. History		Yes	No
**1a**	Ask about anticoagulation use		
**1b**	Ask about the “last known well” time		
**1c**	Ask the patient about his past medical history		
**2**	*Last known well time rationale:* to assess viability of giving thrombolytics within the indicated therapeutic time window		
**II. Physical Examination**			
**3a**	Perform a National Institution of Health Stroke Scale (NIHSS) assessment		
**III. Broad Concepts: Neurologic Assessment**			
**4**	*NIHSS assessment:* As a severity descriptor, to evaluate current neurologic deficits, to track neurologic improvement or decompensation over time		
**IV. Differential Diagnosis**			
**5a**	Ischemic stroke		
**5b**	Differential options: hypoglycemia, acute ischemic stroke, acute hemorrhagic stroke, Todd’s paralysis/seizure, posterior reversible encephalopathy syndrome (PRES), aortic dissection, cervical dissection, transient ischemic attack (TIA), complex migraine, brain mass, recrudescence, cavernous sinus thrombosis.		
**5b**	Differential options: hypoglycemia, acute ischemic stroke, acute hemorrhagic stroke, Todd’s paralysis/seizure, posterior reversible encephalopathy syndrome (PRES), aortic dissection, cervical dissection, transient ischemic attack (TIA), complex migraine, brain mass, recrudescence, cavernous sinus thrombosis.		
**V. Diagnostic Studies**			
**6a**	Point-of-care glucose		
**6b**	CT head		
**6c**	CT angiography brain/neck		
**7**	*Point-of-care glucose rationale:* to evaluate for hypoglycemia as an alternative diagnosis		
**8**	*CT angiography brain/neck rationale:* to evaluate for potential cervical dissection or large vessel occlusion OR NIHSS ≥6 may indicate an LVO		
**VI. Treatment and Other Actions**			
**9a**	Initiate goal blood pressure control to 180–185/105–110		
**9b**	Order thrombolytics **once blood pressure is at goal**		
**10**	*Initiate goal blood pressure control:* to decrease risk of intracerebral hemorrhage after thrombolytics		
**11**	*Order thrombolytics once blood pressure is at goal rationale:* reperfusion to ischemic (but not yet infarcted) areas OR to reduce disability/morbidity		
**VII. Final Diagnosis**			
**12**	Acute ischemic stroke with LVO (must include both components)		
**VIII. Disposition**			
**13**	Admit to the MICU, OR, endovascular suite		
**14**	*Admit location:* For frequent neurologic examinations, MICU, or LVO management for OR/endovascular suite)		
**IX. Transitions of Care**			
**15a**	Explain diagnosis to the patient		
**15b**	*Neurointerventional specialist consultation:* for consideration of thrombectomy.		

**Table:** 

**#1**	**Emergency Department Admitting Form**
**#2**	**Additional Historical Information**
**#3**	**Physical Exam Findings**
**#4**	**Diagnostic Studies**

**Table:** Clinical Decision-Making Candidate Task Sheet

CASE PARAMETERS
This is a 15-minute case You will interact with two examiners. This is an interview style without role playing; you should simply reply to the questions asked. You may be interrupted to move you through the case; this is not a reflection of your performance.
**PATIENT INFORMATION**
Patient is a 45-year-old male who presents with left-sided weakness.
**VITAL SIGNS**
BP: 230/110 mmHg, P: 180/min, R: 30/min, T: 37.0°C, 0_2_ Sat: 98%
**TASK STATEMENT**
Your tasks are as follows: List pertinent elements of a focused history and physical exam Develop an appropriate differential and/or provisional diagnosis Select and interpret appropriate studies Articulate appropriate patient management including discharge instructions

**STIMULUS 1:** Emergency Department Admitting Form

Patient Information	
**Patient Name**	Sam Roberts
**Age**	45-year-old
**Gender**	Male
**Method of Arrival**	Private vehicle
**General Appearance/History of Present Illness**	Patient presents for left-sided weakness.
**Vital Signs on ED Arrival**	BP: 230/110 P: 80 beats per minute R: 30 respirations per minute T: 37° Celsius O2 sat: 98% on room air

**STIMULUS 2:** Additional Historical Information

History of Present Illness/Description of Event	
History of present illness: An hour ago, he first reported a mild headache, then noticed left arm weakness, left leg weakness, and slurred speech. There was no head trauma. No history of strokes, TIAs, or seizures. He has a history of atrial fibrillation on aspirin and hypertension on lisinopril. There is no cocaine use.	
**Past Medical History**	Atrial fibrillation, hypertension
**Past Surgical History**	None
**Medications**	Aspirin, lisinopril
**Allergies**	none
**Social History**	No tobacco, alcohol, or illicit drug use

**STIMULUS 3:** Physical Exam Findings

Physical Examination	
**General**	Patient is conversational but with slurred speech, lying on the cot
**Dermatologic**	normal
**HEENT**	Normal other than left facial droop
**Neck**	Normal
**Respiratory**	Normal
**Cardiac**	Irregular heart rhythm at a regular rate
**Abdominal**	normal
**Extremities**	Normal other than left-sided arm and leg weakness
**Neurologic**	Glascow Coma Scale is 15. Their National Institute of Health Stroke Scale (NIHSS) is 8 (2 dysarthria, 2 left facial droop, 2 left upper extremity (LUE) weakness, 2 left lower extremity (LLE) weakness).

**STIMULUS 4:** Diagnostic Studies

**CBC**	normal
**CMP**	normal
**Glucose**	Normal (100 mg/dL)
**Troponin**	normal
**CT Head** 7	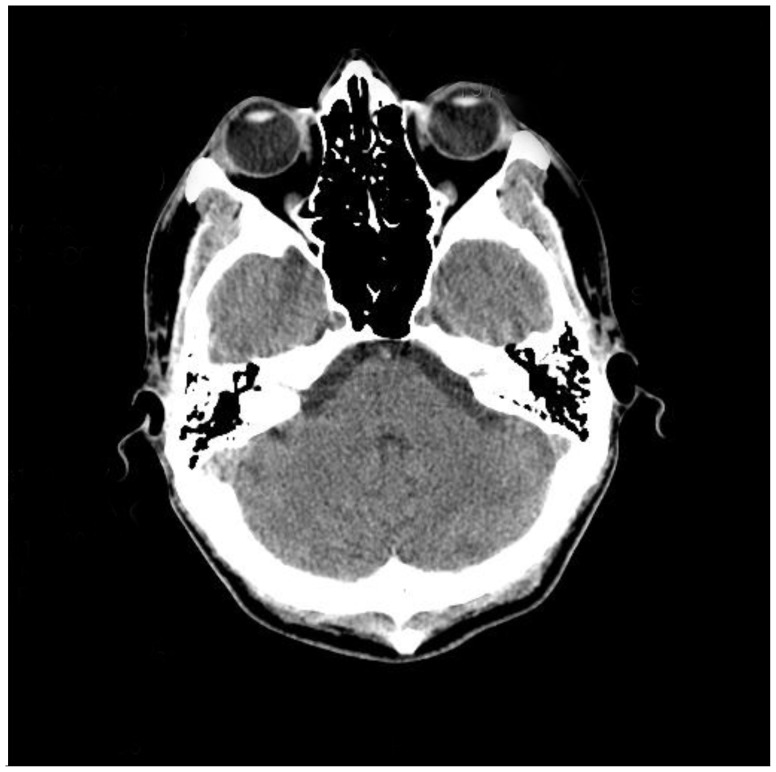
**CT angiography brain and neck**	an acute M2 large vessel occlusion (LVO) is present.

## FOR EXAMINER ONLY

### Clinical Decision-Making Case: Acute Ischemic Stroke with Large Vessel Occlusion

**Diagnosis:** Acute Ischemic Stroke with Large Vessel Occlusion

**Case Summary:** The patient is a 45-year-old male patient who presents with left-sided weakness.

An hour ago, he first reported a mild headache, then noticed left arm weakness, left leg weakness, and slurred speech. There was no head trauma. No history of strokes, transient ischemic attacks (TIAs), or seizures. He has a history of atrial fibrillation on aspirin and hypertension on lisinopril. There is no cocaine use.

### Clinical Decision-Making Case: Acute Ischemic Stroke with Large Vessel Occlusion Examiner Script

**Case Introduction:**

“Hello Doctor, this is a clinical decision-making case. Remember, there is no role playing. You should simply respond to the questions I ask. At times, you may be asked general questions about your clinical decision-making. Additionally, at times I may interrupt you to move you through the case; this is not a reflection of your performance. You will have 15 minutes to complete the case. Do you have any questions? All right, our time begins now.”

“Your patient is a 45-year-old male patient who presents with left-sided weakness.”

Provide Learner Stimulus #1

**HISTORY**

Prompt 1:

“Here is the initial information regarding this patient. After you have read it, please give me a list of the additional historical information you would obtain.”

Scoring Guidelines:

Rationale: Learners should ask about anticoagulation use, the “last known well” time, and past medical history.

Prompt 2a:

“In a patient who presents with lateralizing neurologic deficits, why is it important to ask about the patient’s last known well time?”

Scoring Guidelines:

Rationale: The purpose is to assess viability of giving thrombolytics within the indicated therapeutic time window.

**PHYSICAL EXAMINATION**

“Thank you, doctor, here is the additional historical information.”

Provide Learner Stimulus #2 and read pertinent information.

Prompt 3:

“Now, would you please discuss what you will be assessing on the physical exam of this patient.”

Note for examiner: if any exam stated is not specific enough, you can ask the learner to clarify their “x” exam.

Scoring Guidelines:

Rationale: Learners should perform a neurologic exam to obtain a NIHSS value.

Prompt 4:

“Doctor, why would obtaining a NIHSS number be important?”

Scoring Guidelines:

Rationale: As a severity descriptor, to evaluate current neurologic deficits, to track neurologic improvement or decompensation over time.

“Thank you, doctor; here are the physical exam findings.”

Provide Learner Stimulus #3 and read pertinent information.

**DIFFERENTIAL DIAGNOSIS**

Prompt 5:

“Based on what you now know, what are the top three items on your differential diagnosis based on the most likely conditions?” (If more than three conditions are mentioned, say, “OK, thank you. Please give me your three, and only three, most likely diagnoses.”)

Scoring Guidelines:

Rationale: Stroke MUST be mentioned. Additional differentials can include hypoglycemia, acute ischemic stroke, acute hemorrhagic stroke, Todd’s paralysis/seizure, posterior reversible encephalopathy syndrome (PRES), aortic dissection, cervical dissection, transient ischemic attack (TIA), complex migraine, brain mass, recrudescence, cavernous sinus thrombosis.

**DIAGNOSTIC STUDIES**

Prompt 6:

“Based on what you know and your working differential diagnosis, what, if any, diagnostic studies would you order?”

Scoring Guidelines: Learners should at minimum order a point-of-care glucose, a computed tomography (CT) head, and a CT angiography brain/neck.

Prompt 7:

“Doctor, why would ordering a point-of-care glucose be important in a patient with acute neurologic deficits?

Scoring Guidelines:

Rationale: To assess for hypoglycemia, which is a known cause of acute neurologic deficits and is rapidly reversible with appropriate treatment.

Prompt 8:

“Doctor, in a patient with acute lateralizing neurologic deficits, why may you order a CT angiography of the brain and neck?”

Scoring Guidelines:

Rationale: CT angiography of the brain and neck would allow for evaluation for possible cervical dissection, therefore elucidating the cause of an ischemic stroke OR an NIHSS ≥6 may indicate an large vessel occlusion (LVO).

Provide Learner Stimulus #4: Complete Blood Count (CBC), Complete Metabolic Panel (CMP), point-of-care glucose, troponin, CT head, CT angiography brain/neck.

**TREATMENT AND OTHER ACTIONS**

Prompt 9:

“Based on what you now know, what treatments, if any, would you order and/or what actions, if any, would you perform?”

Scoring Guidelines:

Rationale: Learners should recognize that the blood pressure is too high to safely initiate thrombolytic therapy and should decrease the blood pressure first to a goal of 180–115 systolic blood pressure/105–110 diastolic blood pressure. Once this is achieved, they should order thrombolytic therapy.

Prompt 10:

“Doctor, why are there blood pressure parameters around administering thrombolytics for acute ischemic stroke?”

Scoring Guidelines:

Rationale: To decrease the risk of intracerebral hemorrhage after administering thrombolytics.

Prompt 11:

“Doctor, what is the overall end-goal for administering thrombolytics for acute ischemic stroke?”

Scoring Guidelines:

Rationale: To improve perfusion to the ischemic (but not yet infarcted) penumbra OR to reduce overall morbidity/mortality. If the resident says something along the lines of “to bust up the clot,” they will not get credit.

**FINAL DIAGNOSIS**

Prompt 12:

“Based on everything you know about this case, what is your final diagnosis?”

Scoring Guidelines:

Rationale: Acute ischemic stroke with LVO (needs to include both components). If the candidate mentions something vague such as “stroke” or “cerebrovascular accident [CVA],” the examiner asks, “Can you be more specific about the diagnosis?”

**DISPOSITION**

Prompt 13:

“Based on what you know, what should be the disposition of this patient?”

Scoring Guidelines:

Rationale: Learner should admit the patient to the medical intensive care unit (MICU), operating room (OR), or endovascular suite.

Prompt 14:

“Why would you [admit/discharge] this patient?”

Scoring Guidelines:

Rationale: For frequent neurologic examinations, MICU, or for LVO management (OR/endovascular suite).

**TRANSITION OF CARE**

Prompt 15:

“How could a neuro-intervention specialist (eg, neurosurgery, vascular neurology, interventional radiology) be of use for this patient?”

Scoring Guidelines:

Rationale: To evaluate the patient for a possible thrombectomy, given their large vessel occlusion.

*Thank you, Doctor. That concludes this case.*

*Please tear up your notes*

## CERTIFYING EXAM ASSESSMENT

*Clinical Decision-Making Case: Acute Ischemic Stroke with Large Vessel Occlusion*

Learner: _________________________________________

**Summative and formative comments:**

### Stimulus Inventory

**Candidate Task Sheet**

## DEBRIEFING AND EVALUATION PEARLS

### Clinical Decision-Making Case: Acute Ischemic Stroke with Large Vessel Occlusion

**History**

- Physicians should determine when the “last known well” was for any patient with acute neurologic changes, since this may determine downstream management.
- Patients should be asked about any anticoagulation use such as warfarin, since an INR >1.7 may be a contraindication to thrombolysis.^2^
- A focused history is key, given the time-sensitive nature of some key aspects of stroke management. If a patient has previous neurologic deficits, it should be determined which of the patient’s deficits are new versus baseline.

**Exam**

- If you have a patient that you suspect may have an acute stroke or transient ischemic accident, perform the National Institute of Health Stroke Scale. This will allow for standardization when communicating with your neurology colleagues, as well as for repeat neurologic examinations over time.
- Evaluate for signs of head trauma in those with acute neurologic deficits. If the patient has a history of recent falls and/or signs of head trauma with an intracranial hemorrhage, the patient should undergo a more thorough trauma evaluation.

**Assessment and Plan**

- Order a point-of-care glucose early. Hypoglycemia can cause lateralizing neurologic deficits and is an easily-reversible pathology if treated promptly and appropriately! An untreated glucose <50 mg/dL is a relative contraindication for thrombolytic therapy.^3^
- A Computed Tomography (CT) angiography of the brain and neck can determine if the patient has a causative acute cervical dissection or an acute anterior-circulation large vessel occlusion that is amendable to thrombectomy.
- Traditional treatment windows are within 4.5 hours for intravenous thrombolysis^4^ and within 24 hours for mechanical thrombectomy,^4^ but research in this specific area continues to evolve.
- If thrombolytics are considered, a thrombolytic contraindication list should be reviewed.
- For those receiving thrombolysis, the blood pressure should be treated to a goal below 185/110 millimeters of mercury (mmHg) to reduce the risk of hemorrhagic conversion of ischemic tissue.^2^
- Thrombolysis is thought to protect the penumbra by restoring perfusion to ischemic but not yet infarcted tissue.
- For patients with ischemic stroke due to an acute proximal anterior circulation large vessel occlusion (LVO), endovascular treatment with mechanical thrombectomy devices reduces disability and is superior to treatment with just intravenous thrombolysis. Thrombectomy should occur within 24 hours of the last known well time.^4^ Intravenous thrombolytic therapy for the same ischemic event is not a contraindication.
- Aspirin (160–325 milligrams) should be given within 24–48 hours of symptom onset to reduce the risk of early recurrent ischemic stroke.^5^ If thrombolytics are given, all antiplatelets should be held for 24 hours after thrombolytic therapy if repeat head imaging is negative for hemorrhagic transformation.^6^
